# Cross-Platform Availability of Smartphone Sensors for Depression Indication Systems: Mixed-Methods Umbrella Review

**DOI:** 10.2196/69686

**Published:** 2025-08-07

**Authors:** Johannes Leimhofer, Milica Petrovic, Andreas Dominik, Dominik Heider, Ulrich Hegerl

**Affiliations:** 1 Research Center of German Foundation for Depression and Suicide Prevention Department of Psychiatry, Psychosomatics and Psychotherapy Goethe University Frankfurt Frankfurt am Main Germany; 2 Institute of Computer Science Faculty of Mathematics and Natural Sciences Heinrich Heine University Düsseldorf Düsseldorf Germany; 3 Life Science Informatics Group Department of Mathematics, Natural Sciences and Informatics University of Applied Sciences Giessen Germany; 4 Institute of Medical Informatics University of Münster Münster Germany

**Keywords:** umbrella review, mobile phone, smartphone, sensor data, digital health, depression, cross-platform, data availability

## Abstract

**Background:**

A popular trend in depression forecasting research is the development of machine learning models trained with various types of smartphone sensor data and periodic self-ratings to derive early indications of changes in depression severity. While most works focus on model performance, there is little concern about the universal usability and reliable operation of such systems across smartphone platforms. This review serves as foundational work for the MENTINA clinical trial, which investigates smartphone-based health self-management for depression. The usability and reliability of mobile apps for depression are commonly perceived through the lens of the approaches and interventions offered rather than the reliability of the built-in mobile phone functions to support effortless and exact delivery of intended interventions.

**Objective:**

This work aimed to synthesize existing systematic reviews to identify smartphone sensor modalities used in mental health monitoring and, building on this foundation, assess the cross-platform availability of these data streams using PhoneDB to inform the design and implementation of digital depression indication systems.

**Methods:**

To identify the already used hardware and software sensors and their purposes in mental health monitoring, an umbrella review was conducted. Three electronic databases, including PubMed, Web of Science Core Collection, and Scopus, were searched using smartphone, sensor data, and depression keyword combination to retrieve relevant literature reviews published within the last 5 years (2019-2024). Once the initial search was completed, the extracted hardware sensors were checked for availability on Android and iOS smartphones by analyzing device specifications in PhoneDB over the last 10 years.

**Results:**

The extracted data streams observed across the 9 included studies covered 16 hardware and 3 software data streams. Hardware data streams included accelerometers, barometers, battery levels, Bluetooth, cameras, cellular networks, GPSs, gyroscopes, humidity, light sensors, magnetometers, proximity sensors, sound sensors, step counters, temperature sensors, and Wi-Fi. Software data streams included app usage, call and message logs, and screen status. Hardware component availability on Android and iOS systems showed the changes in component trends from 2014 to 2024 as of September 2024, with the accelerometers, batteries, cameras, and GPSs remaining consistent on Android and iOS, while components such as gyroscopes, step counters, and barometers gradually increased over the years, particularly on Android.

**Conclusions:**

Multiple data streams identified in the literature review showed a consistent increase in availability over time, enabling improved use of these outputs for depression forecasting and the training of machine learning models with diverse smartphone data, including sensor-derived information. For more precise and reliable data to be used in the mental health field, particularly in critical areas such as tracking and predicting changes in depression severity, further research is required to streamline smartphone data across varying mobile hardware and software configurations to provide reliable output for digital mental health purposes.

## Introduction

### Background

The vast variety of personally generated data can provide useful insights that empower individuals with depression to better manage experienced symptoms. Smartphone apps allow for the easy collection of personalized data over long time periods and enable the use of powerful machine learning models that can potentially identify individual data patterns related to the severity of depression or predict changes in symptoms and the probability of relapse or remission. Periodic self-ratings concerning depression-related symptoms and life events are mostly collected actively via questionnaires, whereas data from hardware and software components are mostly recorded passively to derive biomarkers, behavior patterns (eg, phone and app usage) and external moderating factors (eg, environmental conditions).

Several promising works are already using smartphone data to train supervised machine learning models for mental health monitoring [1-6], but only a few are based on continual data from studies lasting longer than 30 days. The existing studies show insufficient consideration of the reliability and practicability of the chosen algorithms in real-world scenarios. Once models are deployed and operated, the performance of model prediction results may vary depending on the algorithm’s capability to handle missing and erroneous data due to reasons such as varying smartphone platforms or data availability. Moreover, the variety of installed hardware and software components and the steadily evolving technology in this field make it even more challenging to build fail-safe applications that work across various environments in the present and future.

Recent literature lists plenty of passively collected smartphone data streams mostly used in a transformed manner for mental health monitoring. While hardware components like accelerometers, ambient light sensors, Bluetooth, cameras, GPSs, gyroscopes, microphones, and Wi-Fi are among the most used smartphone data sources [7-15], hardware components like barometers [8] and temperature [13] sensors are named only a few times in relevant works, but can also play an important role in the context of mental health monitoring. Most sensors are not directly used for depression modeling but rather serve as a proxy for key figures that are correlated with depression symptoms. Typical key figures derived from smartphone hardware components data are estimates on physical activity, sleep duration, sociability, mobility, circadian rhythms, stress, and the environment recognition [7-15]. The use of sensors for deriving key figures varies widely across the literature. For example, sociability has been inferred using microphones, analyzing silence, noise, and voices [14], or by detecting nearby devices via Bluetooth or Wi-Fi [10]. The term “environment” has been applied inconsistently in the literature, encompassing aspects such as an individual’s visual and acoustic surroundings as well as environmental parameters like climatic conditions. For instance, microphones have been used to identify acoustic environments to infer stress [15], while temperature, humidity, and accelerometer sensors have been used to assess environmental hazards [13].

Besides hardware components, smartphone operating systems also provide valuable information for mental health monitoring. Software sensors measuring smartphone and app usage offer key figures on sociability, distractedness, stress, and mood estimates in the case of access permissions to app, phone call, and text message logs [7-15].

Given the presence of multiple existing systematic reviews on smartphone sensing for depression, an umbrella review was chosen to synthesize high-level evidence and critically appraise the quality of these sources—an important step for informing evidence-based mental health app development. By identifying consistently reported sensor modalities and methodological patterns, this review provides a foundation for informed decisions related to recruitment and data collection setup in upcoming clinical studies using smartphone-based mental health monitoring. In particular the results of this work can serve as a comprehensive path for the setting up of the MENTINA trial. The subproject MENTINA trial is part of the European Union–funded research project MENTBEST [16] and aims to investigate the value of data-driven interventions in the context of management of depression severity. Participants of the trial will use an app for 1 year that collects data from smartphone sensors passively while requiring the users to periodically give feedback on personal well-being via self-reports. Besides providing biweekly feedback using the Patient Health Questionnaire 9 [17], participants will also be asked to provide daily ratings on their mental health condition by answering the Patient Health Questionnaire 2 [18] throughout the trial. The app will be available on Android and iOS smartphones and is based on the Monsenso app developed by the Danish digital health solution provider [19-21].

### Objectives

In alignment with the preparation of the MENTINA trial, the following research questions should be answered in the course of this work:

1. Which data streams provided by smartphones have been used in the literature to build models for depression monitoring?
2. Which smartphone data streams are available across smartphone vendors and operating systems to build reliable platform-independent software systems for depression monitoring?
3. Which proxies have been constructed from raw smartphone data streams to build key figures for depression models?

## Methods

### Umbrella Review

#### Protocol and Registration

The umbrella review has been registered in the PROSPERO database (CRD42024581256) and was conducted in accordance with the PRISMA (Preferred Reporting Items for Systematic Reviews and Meta-Analyses) guidelines [22].

#### Information Source and Search Strategy

During the preparation phase of the MENTINA trial in September 2024, the umbrella review was performed by querying the scientific databases PubMed, Web of Science Core Collection, and Scopus to get a comprehensive overview of the existing reviews in the field. The search strategy included querying the scientific databases with the search term “smartphone sensor data depression” to find reviews related to clinical depression or dealing with depression symptoms. To capture the most recent syntheses of evidence on smartphone data streams relevant for current mental health monitoring, filters were applied to include only review papers published within the past 5 years (2019-2024). This time frame aligns with findings that individuals typically replace their smartphones within a 5-year period [23], reinforcing the relevance of focusing on reviews that reflect contemporary device usage and capabilities.

#### Eligibility Criteria

Studies were considered only when the screening of the title and abstract confirmed that the study was a review written in English or German and focused on more than 1 smartphone sensor to build digital phenotypes of individuals’ mental health. The authors JL and MP independently assessed the eligibility of the studies based on titles and abstracts, with UH serving as a supervising rater, resolving any conflicts that arose during the assessment process.

#### Selection of Studies

The full-text versions were obtained and the contents of potentially relevant papers were analyzed to select items matching the following criteria: (1) reviews already summarizing the use of raw smartphone sensors over multiple other works, and (2) reviews showing sensor purposes in terms of depression recognition. The preselection of studies based on the selection criteria was performed by author JL.

#### Quality Assessment

For quality assessment, reviews were evaluated using the AMSTAR (A Measurement Tool to Assess Systematic Reviews) checklist [24]. Reviews were chosen only if particular items could be answered with “yes”. The items of relevance were the following:

- Item 2 (“Was there duplicate study selection and data extraction?”)
- Item 3 (“Was a comprehensive literature search performed?”)
- Item 6 (“Were characteristics of the included studies provided?”)
- Item 11 (“Were potential conflicts of interest included?”).

The full texts were independently quality-assessed by authors UH, MP, and JL. Final inclusion decisions were made through majority consensus.

#### Narrative Synthesis

To synthesize the relevant literature, each included review was screened for smartphone data streams previously used in mental health monitoring. These data streams were extracted and categorized into hardware (derived from installed components) and software (provided by the operating system) categories to distinguish their sources. For each identified data stream, the utilization for deriving mental health insights was assessed and extracted. The extracted data were organized into a table, listing smartphone data streams as rows and relevant studies as columns. Table cells indicated whether a data stream was mentioned in a study regarding mental health monitoring. If a data stream was mentioned but its specific use was unclear, the corresponding cell was marked as “listed.” To enhance readability, similar terms were clustered, and a separate table mapped original terms to umbrella terms. The hardware data stream collection further served as the foundation for investigating cross-platform sensor availability.

The data extraction process was conducted by JL, with MP verifying the extracted data and decisions. UH supervised the process, resolving any conflicts.

### Cross-Platform Sensor Availability

#### Smartphone Operating System Market Analysis

To assess the prevalence of commonly used mobile operating systems, market share data from the past 2 years was analyzed using statistics from the portal Statista [25-29]. To highlight the global significance of Android and iOS, worldwide market share data were extracted. Additionally, regional analyses were focused on Europe, with a particular emphasis on Denmark, Germany, and Spain, to provide insights into the operating system market landscape in these countries where the MENTINA trial will be conducted.

Additionally, to approximate real-world smartphone usage in Germany, the best-selling Android and iOS smartphones in the country for September 2024 were extracted from Counterpoint [30], and the sensor availability in these models was analyzed to better represent the likely scenarios in one of the MENTINA countries.

#### Phone Specifications Database

PhoneDB maintains the world’s largest database for mobile device specifications, providing technical details for smartphones, tablets, smart watches, and several other types of handheld devices [31]. While PhoneDB is not a complete collection, it gives a comprehensive overview of smartphone releases over the past 20 years [32]. PhoneDB was used in a couple of other scientific works [32,33] and serves as a valuable data source for deriving trends for technical specification trends. Besides information on the operating systems the devices are running, the database provides more than 290 parameters per device, including information on the presence of installed hardware sensors. PhoneDB offers licensed database dumps or free limited access to the database via preconfigured database queries using PhoneDB’s web-based detailed parametric search tool. The search tool allows querying device specifications based on built-in sensors, which has been used for this work.

#### Data Queries

PhoneDB was queried multiple times to retrieve the number of devices matching the search criteria. The search criteria included filters on the device category, release date, operating system, and installed hardware including whether the devices are equipped with built-in hardware components. To identify operating system–dependent trends in hardware component availability, the database was queried for annual Android and iOS smartphone specifications with corresponding components installed from 2014 to 2024. To get a baseline, queries without any hardware component filter were executed to get the annual total number of Android and iOS releases for each year.

Additionally, PhoneDB was queried for each considered device to collect pricing information, including price, currency, market countries, and market region, to facilitate analysis of the correlation between price and hardware availability.

#### Data Analysis

The PhoneDB query results were saved in 1 file for iOS and 1 file for Android. Each file consisted of a data table with hardware components as rows and years as columns. The table cells represented the number of device releases in the particular year that had the corresponding hardware component installed. For the baseline, a row consisting of the total number of device releases over the considered years was added to each file. Based on the Android and iOS data tables, the hardware component availability as a percentage was calculated by dividing the number of device releases with the corresponding hardware component installed by the total number of device releases in the respective year and multiplying by 100.

## Results

### Umbrella Review

**Figure 1:**
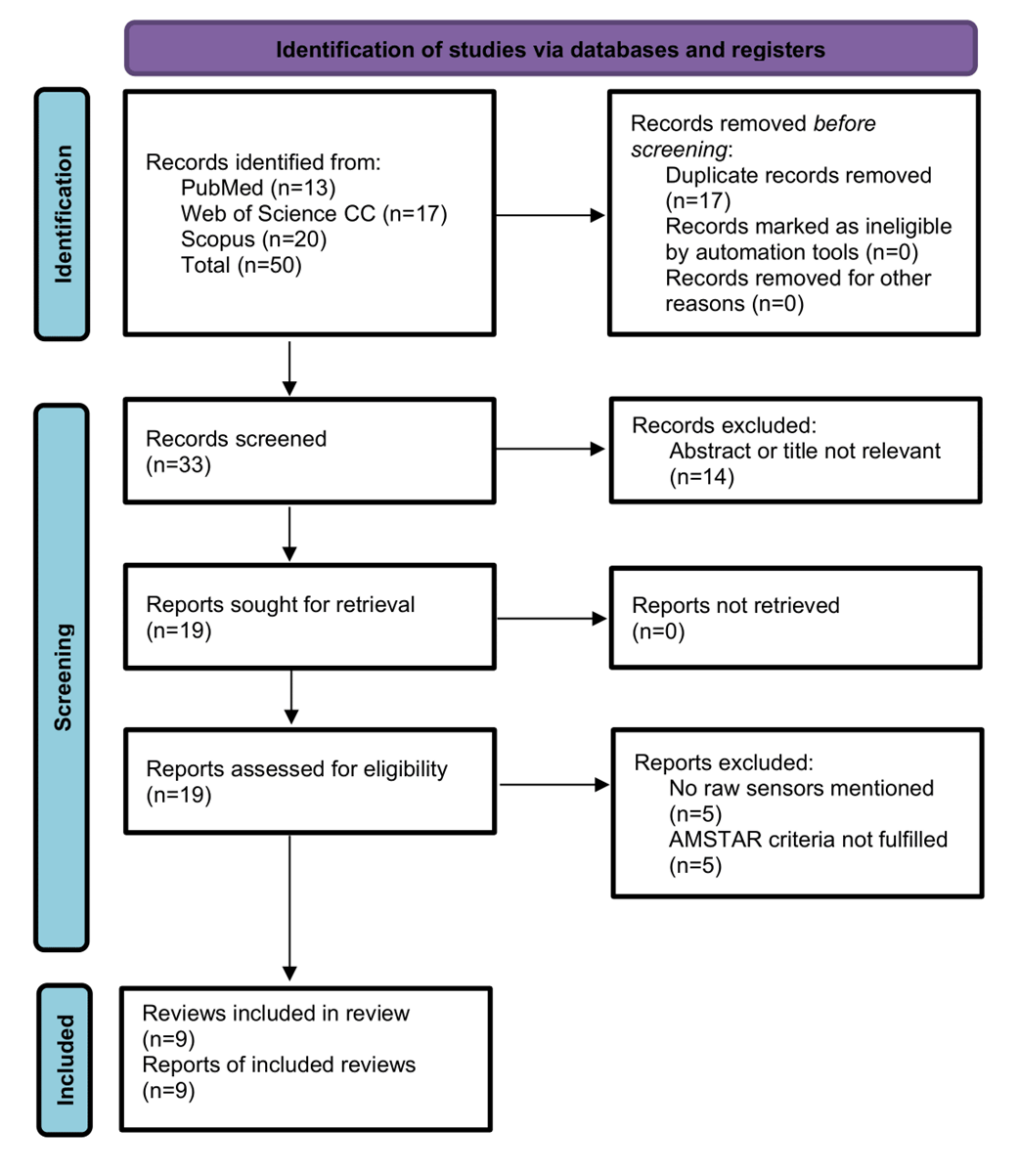
PRISMA (Preferred Reporting Items for Systematic Reviews and Meta-Analyses) flow chart. CC: Core Collection; AMSTAR: A Measurement Tool to Assess Systematic Reviews.

A PRISMA flow chart (Figure 1) has been created to illustrate the information flow of the umbrella review. Aligned with the search strategy, 50 records were found in total across the 3 queried scientific databases (PubMed, n=13; Web of Science Core Collection, n=17; Scopus, n=20). After removing duplicates (n=17), 14 records were removed due to nonrelevant abstract and title. For the remaining 19 records, full-text reports were requested and screened to meet the eligibility criteria. Ten records were excluded for either not reporting the use of raw sensors (n=5) or for not meeting the AMSTAR criteria (n=5) [24]. Finally, 9 reviews were selected for data extraction and synthesis.

The studies included in the umbrella review are listed in Table 1. For transparency, the list of excluded records along with their exclusion reasons can be found in Multimedia Appendix 1. Table 2 depicts the results of the data extraction and synthesis by listing the found smartphone data streams in rows over the relevant studies as columns. The table cells indicate whether a smartphone data stream has been mentioned in the respective article regarding mental health monitoring.

**Table 1:** Included articles in the umbrella review.

Reference	Title
Beames et al [7], 2024	Use of smartphone sensor data in detecting and predicting depression and anxiety in young people (12-25 years): a scoping review
Trifan et al [8], 2019	Passive sensing of health outcomes through smartphones: systematic review of current solutions and possible limitations
Choi et al [9], 2024	Digital phenotyping for stress, anxiety, and mild depression: systematic literature review
Qirtas et al [10], 2022	Loneliness and social isolation detection using passive sensing techniques: scoping review
Yim et al [11], 2020	The utility of smartphone-based, ecological momentary assessment for depressive symptoms
Leaning et al [12], 2024	From smartphone data to clinically relevant predictions: a systematic review of digital phenotyping methods in depression
Virginia Anikwe et al [13], 2022	Mobile and wearable sensors for data-driven health monitoring system: state-of-the-art and future prospect
Lee et al [14], 2023	Using digital phenotyping to understand health-related outcomes: a scoping review
Gopalakrishnan et al [15], 2022	Mobile phone enabled mental health monitoring to enhance diagnosis for severity assessment of behaviours: a review

**Table 2:** Extracted smartphone data streams and their uses in mental health monitoring in each article included in the umbrella review (n=9).

Component		Reference								
		Beames et al [7]	Trifan et al [8]	Choi et al [9]	Qirtas et al [10]	Yim et al [11]	Leaning et al [12]	Virginia Anikwe et al [13]	Lee et al [14]	Gopalakrishnan et al [15]

**Hardware data stream**										
	Accelerometer (n=9)	Physical activity	Physical activity, sleep, and well-being	Physical activity	Physical activity	Circadian rhythm	Physical activity	Physical activity and stress	Mobility, physical activity, and sleep	Physical activity and social activity
	Barometer (n=1)	—^a^	Physical activity	—	—	—	—	—	—	—
	Battery level (n=4)	—	Sleep, well-being, and circadian rhythm	Sleep	—	—	Listed^b^	—	Social activity	—
	Bluetooth (n=8)	Social activity	Circadian rhythm, sleep, social activity, and well-being	Mobility and social activity	Social activity	Social activity	Environment	—	Mobility and social activity	Mobility and social activity
	Camera (n=4)	—	Well-being	Listed	—	Listed	—	—	—	Social activity
	Cellular network (n=3)	—	Listed	—	—	—	Mobility	—	—	Listed
	GPS (n=9)	Mobility	Circadian rhythm, physical activity, sleep, and well-being	Mobility and physical activity	Mobility, physical activity, and social activity	Circadian rhythm	Circadian rhythm, environment, mobility, and physical activity	Circadian rhythm	Mobility	Mobility, physical activity, and social activity
	Gyroscope (n=7)	Listed	Circadian rhythm, physical activity, social activity, and well-being	Physical activity and sleep	—	—	Mobility	Circadian rhythm	Physical activity	Physical activity
	Humidity (n=1)	—	—	—	—	—	—	Well-being	—	—
	Light (n=9)	Sleep	Listed	Sleep	Listed	Listed	Environment and sleep	Listed	Sleep	Sleep and social activity
	Magnetometer (n=2)	—	Physical activity and well-being	—	—	—	—	Circadian rhythm	—	—
	Proximity^c^ (n=1)	—	—	—	—	—	—	Circadian rhythm	—	—
	Sound (n=8)	Social activity	Circadian rhythm, physical activity, sleep, social activity, and well-being	Sleep and social activity	Sleep and social activity	Listed	—	Circadian rhythm	Social activity and sleep	Environment, sleep, and speech
	Step count^d^ (n=6)	Physical activity	Physical activity	Physical activity	—	—	Physical activity	Physical activity	Physical activity	—
	Temperature (n=2)	—	—	—	—	—	—	Physical activity	—	Environment
	Wi-Fi (n=8)	Mobility	Circadian rhythm, physical activity, sleep, social activity, and well-being	Mobility	Social activity	Listed	Mobility	—	Mobility	Mobility, physical activity, and social activity
**Software data stream**										
	App usage (n=8)	Listed	Circadian rhythm, sleep, social activity, and well-being	Social activity	Social activity	Mood	Circadian rhythm, mood, and social activity	—	Social activity	Social activity and stress
	Call and message logs (n=9)	Social activity	Circadian rhythm, sleep, social activity, and well-being	Social activity	Social activity	Mood	Social activity	Circadian rhythm	Social activity and stress	Social activity and stress
	Screen (n=8)	Listed	Listed	Stress and well-being	Listed	Listed	Social activity	—	Social activity and sleep	Sleep

^a^The data stream was not mentioned.

^b^The data stream was mentioned but its specific use was unclear.

^c^Proximity sensors are usually realized with an emitting infrared LED and a detecting photodiode [34].

^d^Step counts are usually derived by sensor fusion of multiple other hardware sensors. Modern smartphones are equipped with dedicated motion coprocessors to do this task in an energy-efficient manner [35].

To improve the readability of Table 2, clusters were built for similar terms. A list of mapped original terms to umbrella terms is shown in Table 3.

**Table 3:** Mapping table of umbrella terms and assigned terms found across the literature.

Umbrella term		Original terms in the literature
**Data stream**		
	App usage	app usage, application usage, browser usage, in-phone activity
	Light	light, ambient light
	Screen	screen status, lock/unlock status, screen time, screen events
	Sound	sound, microphone, audio, voice, speech
**Purpose**		
	Circadian rhythm	circadian rhythm, daily-life behavior, behavioral marker
	Environment	environment, surrounding, workplace conditions
	Mobility	mobility, location, homestay
	Physical activity	physical activity, motion, human activity, movement
	Sleep	sleep, bedtime, sleep disturbance
	Social activity	social activity, loneliness, social avoidance, social rhythm, social function, social habits, social interaction, sociability, social anxiety
	Stress	stress, distractedness

As the results in Table 2 show, the most mentioned hardware data streams included accelerometers, light sensors, and GPSs. While accelerometers are mostly used to derive a proxy measure for physical activity, light sensors are broadly used to gather insights about the sleep behavior of individuals. GPSs serve various purposes to build proxy measures for mental health [7-15]. The most prominent application of GPSs in terms of mental health monitoring is to derive information about individuals’ mobility, which includes, for instance, the number of location changes or the time spent at home. Similar information could be also retrieved from the current position of a smartphone in cellular networks, which was also mentioned in 1 study [12]. In contrast to cellular network information, GPSs were also broadly used to track physical activity and helped to determine one’s circadian rhythm, gathering, for instance, markers for daily life behavior. Only a few studies mentioned GPSs in context of measuring social activity [10,15], but according to the included studies, social activity is often approximated by identifying the number of closely located devices in one’s environment using Bluetooth [7-11,14,15].

Similarly, some of the studies mention the use of microphones or cameras to get a measure for social activity by analyzing individuals’ acoustic and visual environments [7-10,14,15]. Several studies also mention the use of microphones to estimate individuals’ circadian rhythms, including sleep patterns [8-10,13-15]. Those patterns are also approximated by interpreting battery levels, as a couple studies referenced [8,9].

A couple of other hardware data streams were additionally mentioned for estimating circadian rhythms, including data derived from proximity sensors, gyroscopes, or Wi-Fi components [8,13]. Furthermore, Wi-Fi and gyroscopes were mentioned in the context of various other proxy measures for mental health monitoring, including mobility, social activity, and physical activity [7-10,12,14,15].

Beyond the hardware data streams already mentioned, step counts were used many times but exclusively to derive key figures for physical activity [7-9,12-14]. To complete the list of hardware data streams used for representing physical activity, temperature sensors, barometers, and magnetometers were also mentioned in the literature reviews for this purpose [8,13]. Finally, 1 relevant article stated the use of humidity sensors for approximating a person’s well-being [13].

Furthermore, all included studies also listed software data streams for the monitoring of mental health (Table 2), including the data from call and message logs as an insightful data source for building proxy measures for mental health. While most studies stated that call and message logs were used to estimate individuals’ social activity [7-10,12,14,15], 2 referred to using the logs for constructing metrics on circadian rhythm [8,13]. Additionally, estimates for mood and stress were listed as a derivate of call and message logs [11,14,15]. In principle, the same mental health characteristics were derived from using app and screen usage data across the literature, while a few studies stated that screen status was also used to approximate sleep behaviors [14,15].

### Cross-Platform Sensor Availability

According to data from Statista [25], the worldwide smartphone operating system market is dominated by the Android and iOS operating systems, covering 72% and 28% of the market share, respectively, as of the second quarter of 2024. Similarly, the smartphone operating system market in Europe is dominated by Android and iOS [26]. In 2018, Android devices covered 67% of the European market, while the market share for iOS was 32%. In 2023, approximately 65% of European smartphones were still running Android, while the remaining market was almost completely covered by iOS devices. The mobile operating system market shares in Germany, which is part of the MENTINA trial, were similar to those of the broader European market, consisting of 60% Android devices and 39% iOS devices in 2023 [27]. As of March 2024, the mobile operating system market in Spain, another country included in the MENTINA trial, was dominated by Android with a share of approximately 80%, while iOS covered approximately 20% [28]. In contrast, as of April 2024, 57% of mobile devices in Denmark, the third country in the MENTINA trial, were running iOS, while 42% of devices had Android installed [29]. Table 4 summarizes the operating system market shares for the considered regions.

**Table 4:** Smartphone operating system market shares according to Statista [25-29].

	Android (%)	iOS (%)
World	72	28
Europe	65	35
Germany	60	39
Spain	80	20
Denmark	57	42

To estimate real-world smartphone usage in Germany before the start of the MENTINA trial, the top 5 best-selling smartphones in the country for September 2024 were extracted from Counterpoint [30]. The Apple iPhone 15 Pro Max ranked first, accounting for 10% of sales, followed by the Apple iPhone 15 Pro (9%) and the Apple iPhone 16 Pro Max (6%). The list also included Android devices, with the Samsung Galaxy A55 5G ranking fourth (6%) and the Samsung Galaxy S24 ranking fifth (5%).

Due to the worldwide market dominance of Android and iOS smartphones, PhoneDB was only queried for hardware component availability on Android and iOS systems. Table 5 shows the fields and values of PhoneDB’s search tool, combinations of which were used to estimate the trends of hardware component availability for Android and iOS smartphones over the past 10 years.

**Table 5:** Relevant PhoneDB fields and input values to meet the search criteria.

Search criteria			Field		Input values
**General**					
	Year	Release Date		Dates spanning complete years from 2014 to 2024 (eg, from 2020*-*01-01 to 2020-12-31)	
	Smartphone	Device category		Smartphone	
	Operating system	Platform		Android or iOS/iPadOS	
**Data stream**					
	Accelerometer	Built-in accelerometer		Any	
	Barometer	Barometer checkbox		Checked	
	Battery	Battery		Any	
	Bluetooth	Bluetooth		Any	
	Camera	Camera image sensor		Any	
	Cellular network	Supported cellular bands		Any	
	GPS	GPS checkbox		Checked	
	Gyroscope	Built-in gyroscope		Any	
	Humidity	Humidity sensor checkbox		Checked	
	Light	L sensor checkbox		Checked	
	Magnetometer	Built-in compass		Any	
	Microphone	Microphone(s)		Any	
	Proximity	IR^a^ face sensor or LiDAR^b^ checkbox		Checked	
	Step counter	Step counter checkbox		Checked	
	Temperature	T sensor checkbox		Checked	
	Wi-Fi	Wireless LAN^c^		Any	

^a^IR: infrared.

^b^LiDAR: light detection and ranging.

^c^LAN: local area network.

**Figure 2:**
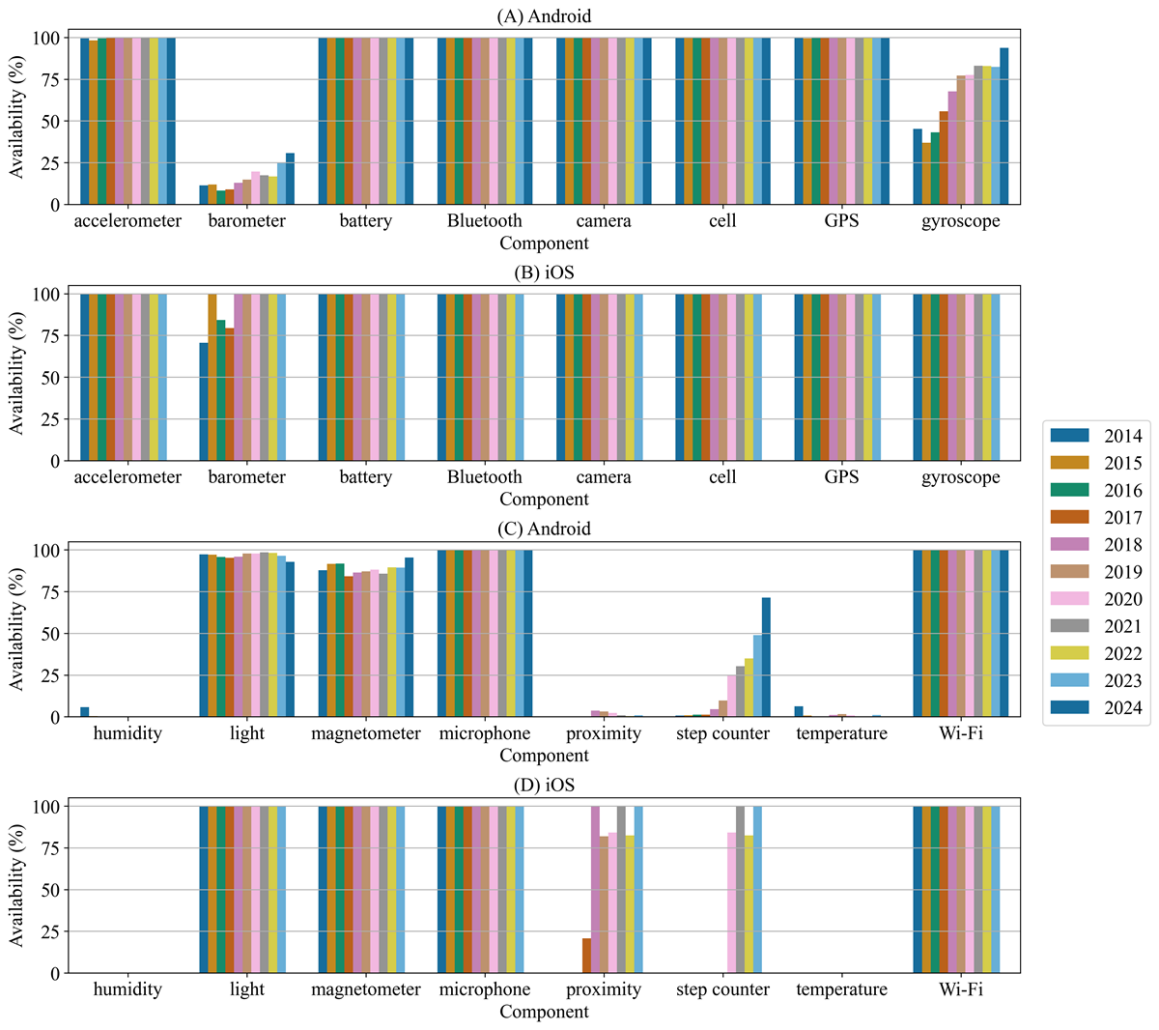
Annual hardware component availability on Android and iOS smartphones from 2014 to 2024 as of September 2024. (A and B) Availability of accelerometer, barometer, battery Bluetooth, camera, cellular network, GPS, and gyroscope components on (A) Android and (B) iOS. (C and D) Availability of humidity, light, magnetometer, microphone, proximity, step counter, temperature, and Wi-Fi components on (C) Android and (D) iOS. The data for each hardware component are plotted in chronological ascending order to depict the availability trend throughout the years. The charts in B and D are missing a bar for 2024 because there were no existing iOS specifications for 2024 as of September 22, 2024.

The raw data derived from the PhoneDB queries can be viewed in Multimedia Appendix 2. The appendix also contains the availability results for Android and iOS. Figure 2 depicts a condensed version of these results by showing the availability of 16 hardware components on Android and iOS smartphones from 2014 to 2024 as of September 2024.

As seen in Figure 2, hardware components such as accelerometers, batteries, Bluetooth, cameras, cellular network support, GPSs, microphones, and Wi-Fi were available on almost every Android and iOS smartphone released in the 10-year period. Since 2018, iOS smartphones have been equipped with barometers. In contrast, only 31% of Android devices came with barometers in 2024, but a positive trend was observed. Similarly, iOS smartphones have provided gyroscopes for the last 9 years, while not all Android devices were shipped with gyroscopes in 2024. With more than 80% of Android smartphones having gyroscopes installed from 2021 to 2023 and approximately 94% in 2024, a positive trend was visible. Besides a few recognizable Android devices in 2014, almost no Android or iOS smartphone provided a humidity sensor in the period studied. Almost the same pattern applied to temperature sensors, with several years seeing low availability on Android systems and no coverage on iOS systems. For light sensors, iOS devices provided full coverage from 2014 to 2023. For Android, 95% of devices provided light sensors from 2014 to 2023 and an all-time low of approximately 93% did so in 2024. Similarly, while magnetometers were found on every iOS smartphone, not every Android device came with magnetometers. The trend of magnetometer availability on Android phones experienced a slow increase from 2017 to 2023, reaching 95% availability in 2024. From 2018 to 2023, iOS smartphones came with a proximity sensor approximately 80% of the time, whereas almost no Android phones provided a proximity sensor during the studied period. Step counters are usually derived by sensor fusion of multiple other hardware sensors, but modern smartphones are equipped with dedicated motion coprocessors to perform this task in a more energy-efficient manner [35]. Coprocessors for efficiently estimating step counts were present on iOS phones more than 80% of the time since 2017; Android systems have rapidly caught up, with an average availability of 71% in 2024.

For transparency, Multimedia Appendix 3 provides a comprehensive list of the smartphones considered in this study, including hardware component availability and price information sourced from PhoneDB. Among the top-selling iOS models, the Apple iPhone 15 Pro Max, iPhone 15 Pro, and iPhone 16 Pro Max include all hardware components listed in Table 5 except for humidity and temperature sensors. In contrast, the top-selling Android models, the Samsung Galaxy A55 5G and Galaxy S24, are equipped with fewer sensors than their iOS counterparts. The more expensive Samsung Galaxy S24 lacks sensors for measuring humidity, proximity, and temperature, while the most affordable among the 5 best-selling models, the Samsung Galaxy A55 5G, is missing additional components, including a barometer, humidity sensor, proximity sensor, step counter, and temperature sensor.

## Discussion

### Principal Results

To highlight the potential of sensors for building reliable mental health monitoring systems across smartphone platforms, we focused on answering three research questions in the course of this work: (1) Which data streams provided by smartphones have been used in the literature to build models for depression monitoring? (2) Which smartphone data streams are available across smartphone vendors and operating systems to build robust platform-independent software systems for depression monitoring? (3) Which proxies have been constructed from raw smartphone data streams to build key figures for depression models?

To address research question (1), an umbrella literature review aligned with the PRISMA workflow was conducted, and 9 articles were included that ensure high methodological quality according to the AMSTAR checklist. From each of the included articles, the mentioned smartphone data streams were extracted, resulting in a list of 16 hardware data streams and 3 software data streams.

To consider research question (3), the purposes of each data stream regarding mental health monitoring were extracted from the literature and assigned to similar purpose clusters. In summary, smartphone data were used in the literature to build key figures for circadian rhythm, the environment, mobility, mood, physical activity, sleep, social activity, speech, stress, and well-being to provide insights into the mental state of individuals.

To provide answers to research question (2), the identified smartphone hardware data streams were assessed regarding their availability on Android and iOS platforms.

In sum, hardware components including accelerometers, batteries, Bluetooth, cameras, cellular network receivers, GPSs, microphones, and Wi-Fi were available on every Android and iOS device released in the past decade. Due to their presence on every smartphone, data streams from these components are promising candidates for the construction of robust cross-platform software systems.

The same applies to data on gyroscopes, light sensors, and magnetometers, which were installed on every iOS and almost every Android smartphone. Despite the fact that step counts are already provided by the operating systems through sensor fusion of other hardware components, the availability of motion coprocessors for more accurate step counters on Android and iOS smartphones increased significantly throughout the studied period, making it another promising candidate for the establishment of robust software systems.

As of September 2024, barometers, humidity sensors, proximity sensors, and temperature sensors had not been consistently installed across Android and iOS mobile phones, and therefore have limited use for reliable cross-platform mental health monitoring systems.

### Limitations

While this work strives to provide a complete picture of the smartphone data available for the development of reliable cross-platform depression indication systems, it does not consider the quality and uniformity of data streams. This work considers a data stream for a particular hardware component to be available if any type of hardware component has been installed. Due to the broad spectrum of installed hardware components in the considered time frame, the data stream signals will vary significantly, thus limiting the comparability and interpretability of raw values across mobile phones. Therefore, future work may focus on improving operating systems and middleware to provide comparable data streams derived from different hardware component types. Some studies have already attempted to address the integration of diverse sensor signals across mobile devices by providing cross-platform frameworks to facilitate the development of stable mobile apps [36,37].

Furthermore, this work lacks an in-depth analysis of how the extracted data streams were specifically used in the literature to compute key metrics for representing mental health. While the studies included in the umbrella review provide a high-level description of the use of smartphone data streams, more detailed research is needed for each data stream individually. An example of in-depth analysis is the investigation of human voice signals derived from smartphones for mood and depression monitoring, which plays a crucial role in the development of mental health monitoring systems [38,39]. Future research should further explore the preprocessing and integration of smartphone data streams to construct representative mental health metrics across platforms.

Although this study primarily focuses on the cross-platform availability of hardware sensors, the umbrella review also identified several software data streams relevant to mental health monitoring, including app usage, call and message logs, and screen status information. However, the included studies do not provide a comprehensive overview of all available data streams offered by operating systems, as they do not account for data accessible through additional operating system functionalities or external application programming interfaces (APIs). For example, a study by Jacobson and Chung [40] illustrates how GPS location data can be enriched with weather conditions by retrieving temperature, humidity, and precipitation data from the National Weather Service API at a specific time and location. Given that humidity and temperature sensors are rarely integrated into Android and iOS smartphones, as indicated by our cross-platform sensor availability analysis, leveraging operating system internal or external APIs could help compensate for these hardware limitations by providing the necessary environmental data. Future research should investigate additional data streams that can be accessed via operating system APIs and external APIs, expanding the range of available information for mental health monitoring.

This umbrella review was designed to support the subsequent cross-platform availability analysis. Given the considerable redundancy observed across the included reviews, a broader search strategy would likely not have materially influenced the main findings, namely the smartphone data streams and their mental health purposes identified in recent years. Nevertheless, the comprehensiveness of the search strategy might be enhanced by the inclusion of additional keyword synonyms. In addition, the time frame for study inclusion was limited to the last 5 years, which may have led to the exclusion of earlier relevant reviews, particularly those covering foundational work or less frequently updated areas. Another limitation concerns the study selection process—while the selection criteria were clearly defined and straightforward to apply, full-text selection was performed by only 1 author (JL), which may have introduced a risk of selection bias. Furthermore, the umbrella review approach, which enabled a high-level synthesis of existing systematic reviews, comes with inherent limitations. As it depends on the scope and quality of the included reviews, any omissions or biases in those sources are carried forward. This may lead to underrepresentation of newer sensor types or recent primary studies not yet covered. Inconsistent reporting across reviews posed a challenge, but relevant information was systematically extracted and harmonized into a unified comparison table, enabling cross-review analysis of sensor modalities and mental health outcomes. Despite these constraints, the method provides a critically appraised foundation to inform future research and application design.

### Comparison With Prior Work

Through the course of this work, plenty of reviews were found that already summarize the potential of smartphone data for mental health monitoring [7-15,41-44]. To our knowledge, there is no umbrella review providing a complete picture of the research in this field. Therefore, this work consolidates the literature findings with respect to the development of cross-platform mental health monitoring systems. The studies of the umbrella review [7-15] mostly highlight the potential of smartphone data for mental health monitoring, but little research has been performed on the availability of underlying data sources across mobile platforms. The availability of data streams is highly dependent on the smartphone operating system [42]. Therefore, this work closes that research gap by providing the availability of smartphone hardware data streams on the predominating smartphone platforms, Android and iOS, over the last 10 years. Vendors and researchers may make use of the availability results to build reliable and robust mental health monitoring software systems in the future.

### Conclusions

Research in the field of digital phenotyping for mental health and depression modeling is an emerging and complex field that integrates insights from psychiatry, technology, and health informatics [45]. Currently, the literature lacks information on passive smartphone data for reliable key figures that represent mental health. This work has started to address this gap by analyzing the cross-platform availability of relevant smartphone data. The results are expected to support the informed decisions concerning the best data collection setup for apps and trials focusing on the value of long-term complex time series analyses of such data in people living with depression and other mental health issues. Nevertheless, further research is needed to streamline smartphone data across varying mobile hardware and software configurations to provide reliable digital mental health for everyone. Therefore, future work may investigate improving frameworks and operating systems to easily provide comparable data streams for mental health monitoring independent of the underlying smartphone.

## Appendix

Multimedia Appendix 1 Studies excluded from the umbrella review and reasons for exclusion.

Multimedia Appendix 2 The full version of the PhoneDB raw data and results of the cross-platform availability analysis.

Multimedia Appendix 3 A comprehensive list of the considered mobile devices for each year including price information as provided by PhoneDB.

## Abbreviations

AMSTAR: A Measurement Tool to Assess Systematic Reviews
API: application programming interface
PRISMA: Preferred Reporting Items for Systematic Reviews and Meta-Analyses
Wi-Fi: Wireless Fidelity

## References

[1] Kathan A, Harrer M, Küster L, Triantafyllopoulos A, He X, Milling M, Gerczuk M, Yan T, Rajamani S, Heber E, Grossmann I, Ebert D, Schuller B (2022). Personalised depression forecasting using mobile sensor data and ecological momentary assessment. Front Digit Health.

[2] Suhara Y, Xu Y, Pentland AS (2017). DeepMood: forecasting depressed mood based on self-reported histories via recurrent neural networks.

[3] Lu J, Shang C, Yue C, Morillo R, Ware S, Kamath J, Bamis A, Russell A, Wang B, Bi J (2018). Joint modeling of heterogeneous sensing data for depression assessment via multi-task learning. Proc ACM Interact Mob Wearable Ubiquitous Technol.

[4] Ware S, Yue C, Morillo R, Lu J, Shang C, Bi J, Kamath J, Russell A, Bamis A, Wang B (2020). Predicting depressive symptoms using smartphone data. Smart Health.

[5] Masud MT, Mamun MA, Thapa K, Lee D, Griffiths MD, Yang S (2020). Unobtrusive monitoring of behavior and movement patterns to detect clinical depression severity level via smartphone. J Biomed Inform.

[6] He X, Triantafyllopoulos A, Kathan A, Milling M, Yan T, Rajamani S, Küster L, Harrer M, Heber E, Grossmann I, Ebert D, Schuller B (2022). Depression diagnosis and forecast based on mobile phone sensor data.

[7] Beames JR, Han J, Shvetcov A, Zheng WY, Slade A, Dabash O, Rosenberg J, O'Dea B, Kasturi S, Hoon L, Whitton AE, Christensen H, Newby JM (2024). Use of smartphone sensor data in detecting and predicting depression and anxiety in young people (12-25 years): a scoping review. Heliyon.

[8] Trifan A, Oliveira M, Oliveira JL (2019). Passive sensing of health outcomes through smartphones: systematic review of current solutions and possible limitations. JMIR Mhealth Uhealth.

[9] Choi A, Ooi A, Lottridge D (2024). Digital phenotyping for stress, anxiety, and mild depression: systematic literature review. JMIR Mhealth Uhealth.

[10] Qirtas MM, Zafeiridi E, Pesch D, White EB (2022). Loneliness and social isolation detection using passive sensing techniques: scoping review. JMIR Mhealth Uhealth.

[11] Yim SJ, Lui LM, Lee Y, Rosenblat JD, Ragguett R, Park C, Subramaniapillai M, Cao B, Zhou A, Rong C, Lin K, Ho RC, Coles AS, Majeed A, Wong ER, Phan L, Nasri F, McIntyre RS (2020). The utility of smartphone-based, ecological momentary assessment for depressive symptoms. J Affect Disord.

[12] Leaning IE, Ikani N, Savage HS, Leow A, Beckmann C, Ruhé HG, Marquand AF (2024). From smartphone data to clinically relevant predictions: a systematic review of digital phenotyping methods in depression. Neurosci Biobehav Rev.

[13] Virginia Anikwe C, Friday Nweke H, Chukwu Ikegwu A, Adolphus Egwuonwu C, Uchenna Onu F, Rita Alo U, Wah Teh Y (2022). Mobile and wearable sensors for data-driven health monitoring system: state-of-the-art and future prospect. Expert Syst Appl.

[14] Lee K, Lee TC, Yefimova M, Kumar S, Puga F, Azuero A, Kamal A, Bakitas MA, Wright AA, Demiris G, Ritchie CS, Pickering CE, Nicholas Dionne-Odom J (2023). Using digital phenotyping to understand health-related outcomes: a scoping review. Int J Med Inform.

[15] Gopalakrishnan A, Venkataraman R, Gururajan R, Zhou X, Genrich R (2022). Mobile phone enabled mental health monitoring to enhance diagnosis for severity assessment of behaviours: a review. PeerJ Comput Sci.

[16] Protecting mental health in times of change. Mentbest.

[17] Kroenke K, Spitzer RL, Williams JB (2001). The PHQ-9: validity of a brief depression severity measure. J Gen Intern Med.

[18] Arrieta J, Aguerrebere M, Raviola G, Flores H, Elliott P, Espinosa A, Reyes A, Ortiz-Panozo E, Rodriguez-Gutierrez EG, Mukherjee J, Palazuelos D, Franke MF (2017). Validity and utility of the Patient Health Questionnaire (PHQ)-2 and PHQ-9 for screening and diagnosis of depression in rural Chiapas, Mexico: a cross-sectional study. J Clin Psychol.

[19] Monsenso. App Store.

[20] Monsenso. Google Play.

[21] Monsenso.

[22] Page MJ, Moher D, Bossuyt PM, Boutron I, Hoffmann TC, Mulrow CD, Shamseer L, Tetzlaff JM, Akl EA, Brennan SE, Chou R, Glanville J, Grimshaw JM, Hróbjartsson A, Lalu MM, Li T, Loder EW, Mayo-Wilson E, McDonald S, McGuinness LA, Stewart LA, Thomas J, Tricco AC, Welch VA, Whiting P, McKenzie JE (2021). PRISMA 2020 explanation and elaboration: updated guidance and exemplars for reporting systematic reviews. BMJ.

[23] Cordella M, Alfieri F, Clemm C, Berwald A (2021). Durability of smartphones: a technical analysis of reliability and repairability aspects. J Clean Prod.

[24] Shea BJ, Hamel C, Wells GA, Bouter LM, Kristjansson E, Grimshaw J, Henry DA, Boers M (2009). AMSTAR is a reliable and valid measurement tool to assess the methodological quality of systematic reviews. J Clin Epidemiol.

[25] Market share of mobile operating systems worldwide from 2009 to 2024, by quarter. Statista.

[26] Market share of leading mobile operating systems in Europe from 2010 to 2023. Statista.

[27] Market share of mobile operating systems in Germany from 2010 to 2023. Statista.

[28] Market share of mobile operating systems in Spain from 2010 to 2024. Statista.

[29] Market share held by the leading mobile operating systems in Denmark as of April 2024. Statista.

[30] Top 5 smartphone models share for 8 countries. Counterpoint.

[31] PhoneDB.

[32] Jost T, Abou Baker N Künstliche intelligenz ermöglicht automatisiertes smartphone-recycling. Prosperkolleg.

[33] Leguesse Y, Colombo C, Vella M, Hernandez-Castro J (2021). PoPL: proof-of-presence and locality, or how to secure financial transactions on your smartphone. IEEE Access.

[34] Rai A, Zhuo D, Bahreyni B (2019). Passive proximity detection based on a miniaturized pyramidal optical sensor.

[35] Lammel G, Dorsch R, Giesselmann T, Goldeck J, Hahn J, Hasan N, Meier J, Gandhi K (2021). Smart system architecture for sensors with integrated signal processing and AI.

[36] Bardram J The CARP mobile sensing framework -- a cross-platform, reactive, programming framework and runtime environment for digital phenotyping. ArXiv. Preprint posted online on June 21, 2020.

[37] Langer P, Altmüller S, Fleisch E, Barata F (2024). CLAID: Closing the Loop on AI & Data Collection — A cross-platform transparent computing middleware framework for smart edge-cloud and digital biomarker applications. Future Gener Comput Syst.

[38] Flanagan O, Chan A, Roop P, Sundram F (2021). Using acoustic speech patterns from smartphones to investigate mood disorders: scoping review. JMIR Mhealth Uhealth.

[39] Shin J, Bae SM (2024). Use of voice features from smartphones for monitoring depressive disorders: scoping review. Digit Health.

[40] Jacobson NC, Chung YJ (2020). Passive sensing of prediction of moment-to-moment depressed mood among undergraduates with clinical levels of depression sample using smartphones. Sensors (Basel).

[41] Majumder S, Deen MJ (2019). Smartphone sensors for health monitoring and diagnosis. Sensors (Basel).

[42] Kulkarni P, Kirkham R, McNaney R (2022). Opportunities for smartphone sensing in e-Health research: a narrative review. Sensors (Basel).

[43] Alamoudi D, Breeze E, Crawley E, Nabney I (2023). The feasibility of using smartphone sensors to track insomnia, depression, and anxiety in adults and young adults: narrative review. JMIR Mhealth Uhealth.

[44] Melcher J, Hays R, Torous J (2020). Digital phenotyping for mental health of college students: a clinical review. Evid Based Ment Health.

[45] Onnela J, Rauch SL (2016). Harnessing smartphone-based digital phenotyping to enhance behavioral and mental health. Neuropsychopharmacology.

