# Supplementary material for: Cross-Platform Availability of Smartphone Sensors for Depression Indication Systems: Mixed-Methods Umbrella Review
Source: Interact J Med Res. 2025 Aug 7;14:e69686. doi: 10.2196/69686 (PMC12371283; doi:10.2196/69686)
Supplement: Multimedia Appendix 1 [file ijmr_v14i1e69686_app1.docx]

| Ref. | Title | Author | Year | Reason for exclusion |
| --- | --- | --- | --- | --- |
| [1] | Smartphone Sensors for Health Monitoring and Diagnosis | Majumder, Deen | 2019 | AMSTAR criteria not fulfilled |
| [2] | Opportunities for Smartphone Sensing in E-Health Research: A Narrative Review | Kulkarni *et al.* | 2022 | AMSTAR criteria not fulfilled |
| [3] | The Feasibility of Using Smartphone Sensors to Track Insomnia, Depression, and Anxiety in Adults and Young Adults: Narrative Review | Alamoudi *et al.* | 2023 | AMSTAR criteria not fulfilled |
| [4] | Digital phenotyping for mental health of college students: a clinical review | Melcher *et al.* | 2020 | AMSTAR criteria not fulfilled |
| [5] | Digital health tools for the passive monitoring of depression: a systematic review of methods | De Angel *et al.* | 2022 | No raw sensors mentioned |
| [6] | Digital phenotyping for assessment and prediction of mental health outcomes: a scoping review protocol | Spinazze *et al.* | 2019 | Abstract/title not relevant |
| [7] | Digital phenotype of mood disorders: A conceptual and critical review | Maatoug *et al.* | 2022 | No raw sensors mentioned |
| [8] | Emerging Artificial Intelligence-Empowered mHealth: Scoping Review | Bhatt *et al.* | 2022 | Abstract/title not relevant |
| [9] | Wearable, Environmental, and Smartphone-Based Passive Sensing for Mental Health Monitoring | Sheikh *et al.* | 2021 | AMSTAR criteria not fulfilled |
| [10] | Use of voice features from smartphones for monitoring depressive disorders: Scoping review | Shin *et al.* | 2024 | Abstract/title not relevant |
| [11] | Smartphone-Delivered Ecological Momentary Interventions Based on Ecological Momentary Assessments to Promote Health Behaviors: Systematic Review and Adapted Checklist for Reporting Ecological Momentary Assessment and Intervention Studies | Dao *et al.* | 2021 | Abstract/title not relevant |
| [12] | Smartphones and the Neuroscience of Mental Health | Gillan, Rutledge | 2021 | Abstract/title not relevant |
| [13] | Using Acoustic Speech Patterns From Smartphones to Investigate Mood Disorders: Scoping Review | Flanagan *et al.* | 2021 | Abstract/title not relevant |
| [14] | A survey of autonomous monitoring systems in mental health | Gopalakrishnan *et al.* | 2024 | No raw sensors mentioned |
| [15] | Digital Phenotyping and Patient-Generated Health Data for Outcome Measurement in Surgical Care: A Scoping Review | Jayakumar *et al.* | 2020 | Abstract/title not relevant |
| [16] | Ecological momentary interventions for mental health: A scoping review | Balaskas *et al.* | 2021 | Abstract/title not relevant |
| [17] | Remote sensing mental health: A systematic review of factors essential to clinical translation from validation research | Bidargaddi *et al.* | 2024 | No raw sensors mentioned |
| [18] | Wearable devices for anxiety & depression: A scoping review | Ahmed *et al.* | 2023 | Abstract/title not relevant |
| [19] | Digital Devices for Assessing Motor Functions in Mobility-Impaired and Healthy Populations: Systematic Literature Review | Guo *et al.* | 2022 | Abstract/title not relevant |
| [20] | Implementing Digital Technologies in Clinical Trials: Lessons Learned | Mitsi *et al.* | 2022 | Abstract/title not relevant |
| [21] | Telemonitoring of real-world health data in cardiology: A systematic review | Kinast *et al.* | 2021 | Abstract/title not relevant |
| [22] | Digital Health Around Clinical High Risk and First-Episode Psychosis | Henson *et al.* | 2020 | Abstract/title not relevant |
| [23] | Precision Medicine and Suicide: an Opportunity for Digital Health | Barrigon *et al.* | 2019 | No raw sensors mentioned |
| [24] | Remote assessment of disease and relapse in major depressive disorder (RADAR-MDD): a multi-centre prospective cohort study protocol | Matcham *et al.* | 2019 | Abstract/title not relevant |

1. Majumder S, Deen MJ. Smartphone Sensors for Health Monitoring and Diagnosis. Sensors Multidisciplinary Digital Publishing Institute; 2019 Jan;19(9):2164. doi: 10.3390/s19092164

2. Kulkarni P, Kirkham R, McNaney R. Opportunities for Smartphone Sensing in E-Health Research: A Narrative Review. Sensors Multidisciplinary Digital Publishing Institute; 2022 Jan;22(10):3893. doi: 10.3390/s22103893

3. Alamoudi D, Breeze E, Crawley E, Nabney I. The Feasibility of Using Smartphone Sensors to Track Insomnia, Depression, and Anxiety in Adults and Young Adults: Narrative Review. JMIR MHealth UHealth 2023 Feb 17;11(1):e44123. doi: 10.2196/44123

4. Melcher J, Hays R, Torous J. Digital phenotyping for mental health of college students: a clinical review. Evid Based Ment Health 2020 Nov;23(4):161–166. PMID:32998937

5. De Angel V, Lewis S, White K, Oetzmann C, Leightley D, Oprea E, Lavelle G, Matcham F, Pace A, Mohr DC, Dobson R, Hotopf M. Digital health tools for the passive monitoring of depression: a systematic review of methods. Npj Digit Med Nature Publishing Group; 2022 Jan 11;5(1):1–14. doi: 10.1038/s41746-021-00548-8

6. Spinazze P, Rykov Y, Bottle A, Car J. Digital phenotyping for assessment and prediction of mental health outcomes: a scoping review protocol. BMJ Open British Medical Journal Publishing Group; 2019 Dec 1;9(12):e032255. PMID:31892655

7. Maatoug R, Oudin A, Adrien V, Saudreau B, Bonnot O, Millet B, Ferreri F, Mouchabac S, Bourla A. Digital phenotype of mood disorders: A conceptual and critical review. Front Psychiatry 2022 Jul 26;13:895860. PMID:35958638

8. Bhatt P, Liu J, Gong Y, Wang J, Guo Y. Emerging Artificial Intelligence-Empowered mHealth: Scoping Review. JMIR MHealth UHealth 2022 Jun 9;10(6):e35053. PMID:35679107

9. Sheikh M, Qassem M, Kyriacou PA. Wearable, Environmental, and Smartphone-Based Passive Sensing for Mental Health Monitoring. Front Digit Health 2021;3:662811. PMID:34713137

10. Shin J, Bae SM. Use of voice features from smartphones for monitoring depressive disorders: Scoping review. Digit Health 2024;10:20552076241261920. PMID:38882248

11. Dao KP, De Cocker K, Tong HL, Kocaballi AB, Chow C, Laranjo L. Smartphone-Delivered Ecological Momentary Interventions Based on Ecological Momentary Assessments to Promote Health Behaviors: Systematic Review and Adapted Checklist for Reporting Ecological Momentary Assessment and Intervention Studies. JMIR MHEALTH UHEALTH Toronto: Jmir Publications, Inc; 2021 Nov 2;9(11):e22890. doi: 10.2196/22890

12. Gillan CM, Rutledge RB. Smartphones and the Neuroscience of Mental Health. In: Roska B, Zoghbi HY, editors. Annu Rev Neurosci VOL 44 2021 Palo Alto: Annual Reviews; 2021. p. 129–151. doi: 10.1146/annurev-neuro-101220-014053ISBN:978-0-8243-2444-5

13. Flanagan O, Chan A, Roop P, Sundram F. Using Acoustic Speech Patterns From Smartphones to Investigate Mood Disorders: Scoping Review. JMIR MHEALTH UHEALTH Toronto: Jmir Publications, Inc; 2021 Sep;9(9):e24352. doi: 10.2196/24352

14. Gopalakrishnan A, Gururajan R, Zhou X, Venkataraman R, Chan KC, Higgins N. A survey of autonomous monitoring systems in mental health. WILEY Interdiscip Rev-DATA Min Knowl Discov San Francisco: Wiley Periodicals, Inc; 2024 May;14(3). doi: 10.1002/widm.1527

15. Jayakumar P, Lin E, Galea V, Mathew AJ, Panda N, Vetter I, Haynes AB. Digital Phenotyping and Patient-Generated Health Data for Outcome Measurement in Surgical Care: A Scoping Review. J Pers Med Basel: MDPI; 2020 Dec;10(4):282. doi: 10.3390/jpm10040282

16. Balaskas A, Schueller SM, Cox AL, Doherty G. Ecological momentary interventions for mental health: A scoping review. PLoS ONE San Francisco: Public Library Science; 2021 Mar 11;16(3):e0248152. doi: 10.1371/journal.pone.0248152

17. Bidargaddi N, Leibbrandt R, Paget TL, Verjans J, Looi JC, Lipschitz J. Remote sensing mental health: A systematic review of factors essential to clinical translation from validation research. Digit Health SAGE Publications Ltd; 2024 Jan 1;10:20552076241260414. doi: 10.1177/20552076241260414

18. Ahmed A, Aziz S, Alzubaidi M, Schneider J, Irshaidat S, Abu Serhan H, Abd-alrazaq AA, Solaiman B, Househ M. Wearable devices for anxiety & depression: A scoping review. Comput Methods Programs Biomed Update 2023;3. doi: 10.1016/j.cmpbup.2023.100095

19. Guo CC, Chiesa PA, de Moor C, Fazeli MS, Schofield T, Hofer K, Belachew S, Scotland A. Digital Devices for Assessing Motor Functions in Mobility-Impaired and Healthy Populations: Systematic Literature Review. J Med Internet Res 2022;24(11). doi: 10.2196/37683

20. Mitsi G, Grinnell T, Giordano S, Goodin T, Sanjar S, Marble E, Pikalov A. Implementing Digital Technologies in Clinical Trials: Lessons Learned. Innov Clin Neurosci 2022;19(4–6):65–69.

21. Kinast B, Lutz M, Schreiweis B. Telemonitoring of real-world health data in cardiology: A systematic review. Int J Environ Res Public Health 2021;18(17). doi: 10.3390/ijerph18179070

22. Henson P, Wisniewski H, Stromeyer IV C, Torous J. Digital Health Around Clinical High Risk and First-Episode Psychosis. Curr Psychiatry Rep 2020;22(11). doi: 10.1007/s11920-020-01184-x

23. Barrigon ML, Courtet P, Oquendo M, Baca-García E. Precision Medicine and Suicide: an Opportunity for Digital Health. Curr Psychiatry Rep 2019;21(12). doi: 10.1007/s11920-019-1119-8

24. Matcham F, Barattieri di San Pietro C, Bulgari V, de Girolamo G, Dobson R, Eriksson H, Folarin AA, Haro JM, Kerz M, Lamers F, Li Q, Manyakov NV, Mohr DC, Myin-Germeys I, Narayan V, BWJH P, Ranjan Y, Rashid Z, Rintala A, Siddi S, Simblett SK, Wykes T, Hotopf M, DiFrancesco S, White K, Ivan A, Polhemus A, Ferrao J, Ringkjøbing-Elema M, Nobilia F, Viechtbauer W, Peelen S, Rashid Z, Boere J, Cummins N, Meyer N, on behalf of the RADAR-CNS consortium. Remote assessment of disease and relapse in major depressive disorder (RADAR-MDD): a multi-centre prospective cohort study protocol. BMC Psychiatry 2019 Feb 18;19(1):72. doi: 10.1186/s12888-019-2049-z
